# Biodeterioration of Compost-Pretreated Polyvinyl Chloride Films by Microorganisms Isolated From Weathered Plastics

**DOI:** 10.3389/fbioe.2022.832413

**Published:** 2022-02-10

**Authors:** Čenek Novotný, Jindřich Fojtík, Martin Mucha, Kateřina Malachová

**Affiliations:** ^1^ Laboratory of Environmental Biotechnology, Institute of Microbiology of the Czech Academy of Sciences, Prague, Czech Republic; ^2^ Department of Horticulture, Faculty of Agrobiology, Food and Natural Resources, Czech University of Life Sciences Prague, Prague, Czech Republic; ^3^ Institute of Environmental Technology, CEET, VSB-Technical University of Ostrava, Ostrava, Czech Republic; ^4^ Department of Biology and Ecology, Faculty of Science, University of Ostrava, Ostrava, Czech Republic; ^5^ Department of Chemistry, Faculty of Science, University of Ostrava, Ostrava, Czech Republic

**Keywords:** polyvinyl chloride, biodeterioration, composting, SSF culture, Trichoderma hamatum, Bacillus amyloliquefaciens

## Abstract

Polyvinyl chloride (PVC) is a petroleum-based plastic used in various applications, polluting the environment because of its recalcitrance, large content of additives, and the presence of halogen. In our case study, a new, two-stage biodegradation technology that combined composting process used for PVC pretreatment with a subsequent PVC attack by newly-isolated fungal and bacterial strains under SSF conditions was used for biodegradation of commercial PVC films. The novelty consisted in a combined effect of the two biodegradation processes and the use for augmentation of microbial strains isolated from plastic-polluted environments. First, the ability of the newly-isolated strains to deteriorate PVC was tested in individual, liquid-medium- and SSF cultures. Higher mass-reductions of PVC films were obtained in the former cultures, probably due to a better mass transfer in liquid phase. Using the two-stage biodegradation technology the highest cumulative mass-reductions of 29.3 and 33.2% of PVC films were obtained after 110 days with *Trichoderma hamatum* and *Bacillus amyloliquefaciens* applied in the second stage in the SSF culture, respectively. However, FTIR analysis showed that the mass-reductions obtained represented removal of significant amounts of additives but the PVC polymer chain was not degraded.

## Introduction

The accumulation of plastics in the environment represents a great problem concerning the long-term waste management. PVC, one of the most often used petroleum-based plastic polymers whose European year demand was about 5 million tons in 2019, is used for food packaging, electronics coatings, medical devices and other industrial applications (Plastics Europe, 2021). Plasticized PVCs contain additives, most often phthalate esters, that improve mechanical properties and durability of the plastic and can represent up to 50% W/W (Ru et al., 2020). Phthalates have been recognized to be endocrine disruptors with genotoxic effects, but other components, such as heavy metals and chlorine atoms, are also of environmental concern in connection with PVC deterioration in the environment and the disposal in landfills (Glas et al., 2014; Wen et al., 2014; Al-Saleh et al., 2017; Siciňska et al., 2021).

The PVC resistance to biodegradation under the conditions in the environment was well documented by reporting no evidence of degradation of a PVC cable coating buried in soil for 32–37 years (Otake et al., 1995) as well as by other studies (Khatoon et al., 2019; Kumari et al., 2019). An extensive research has been undertaken to specify microorganisms and their consortia capable of degrading PVC, including those colonizing plastic waste (e.g. Webb et al., 2000; Patil and Bagde, 2012; Giacomucci et al., 2020; Malachová et al., 2020). Measured gravimetrically, various weight losses values were reported by different authors, for instance, 3–14% after a 28-month exposure to a consortium of microoganisms from biowaste fermentation plant applied under landfill simulation conditions (Mersiowsky et al., 2001), 1.8–6.8% (W/W) after a 6-week exposure to 20 different fungal strains isolated from PVC surface (Webb et al., 2000), 13–19% for PVC films incubated with *Pseudomonas citronellolis* for 30 days (Giacomucci et al., 2019), or 2–13% for PVC films incubated for 24 months with 16 different anaerobic microcosms enriched from marine samples (Giacomucci et al., 2020). However, the microorganisms mentioned above metabolized predominantly plasticizer molecules present in PVC rather than the PVC polymer chain.

The predominant plasticizers used for PVC plastics are phthalic esters, other types of plasticizers include molecules such as carboxylic acid esters, epoxides, polyesters or, more recently, renewable resource-based plasticizers (OSPARCOM, 1997; Mukherjee and Ghosh, 2019). A vast number of bacteria and fungi were reported to degrade phthalates under aerobic and anaerobic conditions and the degradation pathways were described. The enzymes involved are esterases, decarboxylases and various redox active enzymes, dioxygenases and dehydrogenases (Liang et al., 2008; Ahuactzin-Perez et al., 2018; Zhang et al., 2018; Khatoon et al., 2019). The biodegradation of phthalates involves a sequential hydrolysis of the ester linkage and, subsequently, of phthalic acid. Microbial assimilation of phthalates requires diverse metabolic genes and enzymes and, thus, microbial consortia are much more efficient in mineralization of phthalates (Wang et al., 2004; Chatterjee and Dutta, 2008).

The attacks on the PVC polymer were reported in the case of *P. citronellolis* and several anaerobic consortia proven by TGA and GPC analyses, the GPC analysis showed a reduction in average molecular weight of PVC of 10% in the case of *P. citronellolis* (Giacomucci et al., 2019, 2020). Similarly, fungi of the genera Penicillium and Mucor were reported to attack PVC films (Pardo-Rodríguez and Zorro-Mateus, 2021). Patil and Bagde (2012) reported that a *Micrococcus* sp. strain isolated from a plastic materials-polluted site showed a 0.36% release of chloride and 8.9% mineralization measured in terms of CO_2_ evolution over a period of 70 days in media containing PVC as a sole carbon source. The enzymes involved in the microbial degradation of PVC are unknown (Ru et al., 2020). Recently, Peng et al. (2020) showed that *Tenebrio molitor* larvae were able to depolymerize PVC (M_n_ 82,2, M_w_ 143,7, Mz 244.9 kDa) probably containing only traces of PAEs and brominated flame retardants and this process was gut microbe-dependent with the operational taxonomic unit of *Lactococcus* bacteria being suspected to be the functional microbe species.

Solid-state fermentation (SSF) is a method of growing microorganisms in a solid-phase substrate containing low amounts of free water used for production of biomolecules, treatment of organic wastes, or in organopollutant-remediation applications (Thomas et al., 2013). The conditions are well suited especially for fungal organisms that can produce their extracellular enzyme activities and use them for decomposition of lignocellulosic substrates and organopollutants, but bacteria can also be used in those technologies (Couto and Sanroman, 2006; Chang et al., 2018). The efficiency of the SSF method for bioremediation of solid substrates and soils contaminated with recalcitrant organopollutants, e.g. industrial dyes, bisphenol A, dioxins, PAHs, as well as for degradation of biodegradable plastics has been widely proven (Zeng et al., 2017; Folino et al., 2020; Kaewlaoyoong et al., 2020; Vipotnik et al., 2021).

The introduction of hydroxyl- and carbonyl groups in the recalcitrant polymer molecule is essential for the splitting of the polymer chain by extracellular enzymes to small fragments that can subsequently enter the intracellular metabolism to serve as carbon and energy sources for growth (Arutchelvi et al., 2008). Such groups can be formed, for instance, by a pretreatment procedure including a combination of γ-irradiation and high-temperature treatments, as demonstrated for the LLDPE polymer (Novotny et al., 2018). Other pretreatment methods include photooxidation, UV treatment, thermal aging or an outdoor weathering process (Chiellini et al., 2006; Briassoulis et al., 2015; Anunciado et al., 2021). Colonization of PE materials surfaces by bacteria and fungi was found to be the first stage of a microbial attack by changing the surface appearance and making it more hydrophilic. Simultaneously, changes of carbonyl index resulting from the formation of ketone or aldehyde groups that can further undergo β-oxidation to produce CO_2_ and water were observed (Gu, 2007; Sudhakar et al., 2008; Nowak et al., 2011). Composting process is an exothermic biooxidation in which the organic substrate is biodegraded by a mixed population of microorganisms, including bacteria, archaea, and fungi (Nozhevnikova et al., 2019). It has a large remediation potential for degradation and removal of various persistant pollutants, such as petroleum hydrocarbons, polyaromatic hydrocarbons, dioxin, and pharmaceuticals, from contaminated soil, sewage sludge and other waste materials (Iranzo et al., 2018; Guo et al., 2020; Tran et al., 2020; Tran et al., 2021). Composting process can accelerate biodegradation of additives, for instance phthalic acid esters (PAEs) and polyhydroxyalkanoates (PHAs), and thus reduce mass of PVC film (Amir et al., 2005; Chang et al., 2009; Anunciado et al., 2021). The surface of PVC and PHA microplastics exposed to a composting process was found to be eroded, their carbon contents decreased, and the amount of functional O-H, C=O, and C-O groups increased after the compost treatment (Sun et al., 2021). The effect of compost is in this aspect similar to physico-chemical pretreatment processes (Martinez et al., 2020).

Because of the PVC recalcitrance the research perspective concerning PVC biodegradation should focus on both an efficient pretreatment resulting in massive formation of functional groups on PVC surface and on finding new strains and consortia of microorganisms possessing enzyme activities capable of depolymerization of PVC. Maximization of plastic surface exposed to biodegradation, for instance by using PVC microparticles, can also be an important factor (Giacomucci et al., 2020; Peng et al., 2020). The aim of our case study was to investigate the efficiency of a newly-developed, two-stage biodegradation process combining the effect of composting with a subsequent SSF degradation by fungal and bacterial strains isolated from plastic-polluted environments. The novelty consisted in using the composting process for the pretreatment of PVC followed by application of new isolated, fungal and bacterial strains for PVC degradation. To our knowledge such a combined technology has so far not been used for the degradation of PVC. The biodegradation of PVC in the two-stage process was measured gravimetrically and using FTIR analysis, the biodeterioration of PVC was also documented by SEM. The results were compared with PVC weight losses obtained by exposure to the isolated fungal and bacterial strains applied in a liquid growth medium and in SSF cultures under aseptic conditions.

## Materials and Methods

### Microorganisms and Material

The following strains of microorganisms were used in the case study: the fungi *Trichoderma hamatum* HF4 (origin: plastics from soil along a highway, Belgium), *Trichaptum abietinum* CA (origin: plastics from a composting plant, Schendelbeke, Belgium), *Pseudoallescheria ellipsoidea* ZF51 (origin: plastics from a composting plant, Schendelbeke, Belgium), *Aspergillus terreus* CCF 3315 (origin Czech Collection of Fungi), and the bacterium *Bacillus amyloquefaciens* JB4 (origin: plastics from a composting plant, Schendelbeke, Belgium). Except for *A. terreus* CCF 3315, they were isolated from weathered plastics in the environment and characterized in a previous study of Malachová et al. (2020). Other strains also isolated from the surface of plastics collected in the environment whose origin and taxonomical characterization are described in detail by Malachova et al. (2020), namely *Byssochlamys nivea* JM5, *Pseudoallescheria ellipsoidea* ZF51, *Aspergillus coesiellus* ZB52, *Graphium* sp. ZF56, *Trametes suaveolens* F1, *Penicillium olsonii* ZF52, *Scopulariopsis brevicaulis* ZF53. The GenBank Accession Nos. of all the isolated strains used in this study were published by Malachova et al. (2020). *Penicillum variabile* CCF3319 and *Aspergillus terreus* CCF 3315 were obtained from Czech Collection of Fungi, Charles University, Prague. *Rhodococcus ruber* C208 strain (seawater isolate, Norway) was obtained from Fachhochschule Nordwestschweiz, Muttenz, Switzerland.

The microorganisms were stored on MEG agar slants (per litre: malt extract 20 g, glucose 20 g, peptone 1 g, agar 20 g, pH 5.4) at 4°C.

PVC films were produced by Gruppo Fabbri Spa. (Italy). They contained plasticizers whose formulation was confidential.

### PVC Degradation in Liquid Growth Media Under Aseptic Conditions

Before used in a two-stage degradation process, the fungal strains were tested for the ability to attack PVC films in liquid Czapek-Dox medium, pH 6.8 (g/L: sucrose 30, sodium nitrate 2, dipotassium phosphate 1, magnesium sulfate 0.5, potassium chloride 0.5, ferrous sulfate 0.01) and the bacteria in Bushnel-Haas medium, pH 7.0 (g/L: magnesium sulfate 0.2, calcium chloride 0.02, potassium hydrogenphosphate 1, potassium dihydrogenphosphate 1, ammonium nitrate 1, ferric chloride 0.05, glucose 5, Fluka, Germany). The aim was to select efficient and robust strains for the case study experiments. The protocol used in the biodegradation experiments was the same as described by Novotný et al*.* (2018). Before the treatment, the films were cut into small pieces (20–30 mg) and degreased by immersion in 70% ethanol for 10 min at 120 rpm (ELMI orbital shaker DOS-20L, ELMI Ltd., Latvia). Plastic samples were weighed after drying and sterilized in 70% ethanol for 30 min at 120 rpm. By rinsing in sterile distilled water the samples were prepared for experiments (Peixoto et al., 2017).

The biodegradation by fungal strains was carried out aseptically in liquid Czapek Dox medium, pH 6.8. The inoculum was prepared by growing the fungi statically in MEG medium pH 4.5 (g/L: malt extract 5, glucose 10) for 7 days, gently homogenized (UltraTurrax T25, IKA Labortechnik, Germany) and used at 5% (V/V) for inoculation of 500-ml erlenmeyer flasks containing 100 ml of the medium. After inoculation, the cultures were incubated statically (stirred manually every other day) at 28°C for 2 months, aerated by air diffusion. The fungal precultures in MEG medium were inoculated from fresh agar plate cultures.

The biodegradation by *B. amyloliquefaciens* occurred under aseptic conditions in liquid Bushnel-Haas medium pH 7.0 (Fluka, Germany) enriched with 1 g/L glucose. The medium does not contain any carbon source and, thus, a small amount of glucose was added to support the cometabolic process and maintain the bacterial culture viable and active during the 2-month experiment. The aeration was ensured by shaking at 120 rpm using an orbital shaker. The bacterial inoculum (5%, V/V) was prepared by overnight growth in a shaken culture at 28°C using Boyd-Kohlmeyer medium pH 6.8 (g/L: glucose 10, peptone 2, yeast extract 1).

When the cultivation was terminated, the plastic samples were collected and microbial biofilm removed from their surface by immersion in 2% SDS for 2 h at 50°C, followed by a 15-min ultrasonication, then the PVC films were rinsed with distilled water and immersed in 70% ethanol, vortexed and finally washed with distilled water and dried. This procedure was repeated five times (Sivan et al., 2006) and was used in all biodegradation experiments. The degradation was measured gravimetrically (ABT 220-5DM, KERN, Germany), after drying. Each experiment was conducted in triplicates and always included an abiotic control.

Sterilization of all media was by autoclaving (120°C, 20 min) (Systec VX-5, Systec GmbH, Germany).

### PVC Degradation in Laboratory-Scale SSF Cultures Under Aseptic Conditions

The SSF culture used in the laboratory-scale, solid-state degradation experiments consisted of wheat bran flakes (500 g) moisturized with a salt solution (pH 5.0) containing (per L) ammonium nitrate 4.3 g, sodium sulfate 0.21 g, magnesium sulfate 0.77 g, zinc sulfate 0.42 g, potassium chloride 1.62 g, and calcium hydroxide 0.11 g (Rodríguez-Fernández et al., 2012) to reach 50–60% moisture. PVC films were cut into 20–40 mg pieces, degreased with 70% ethanol and dried overnight. They were weighed and sterilized in 70% ethanol for 30 min, washed with sterile distilled water and dried. Then they were wrapped in a nylon mesh (16 × 8 cm, 0.5 × 0.5 cm pore size) and inserted inside the solid phase layer. The system was inoculated with a volume of 150 ml of a homogenized, 7-day-old culture grown in liquid MEG medium at 28°C. The initial moisture of the solid phase was 50–60% and was kept constant during the experiment by adding sterile distilled water. The duration of the experiment was 2 months, the temperature was maintained between 24 and 28°C. The aeration was by air diffusion and ensured by regular manual turning once a week.

### Two-Stage Pilot-Scale, Aerobic Degradation of PVC Films

**FIGURE 1:**
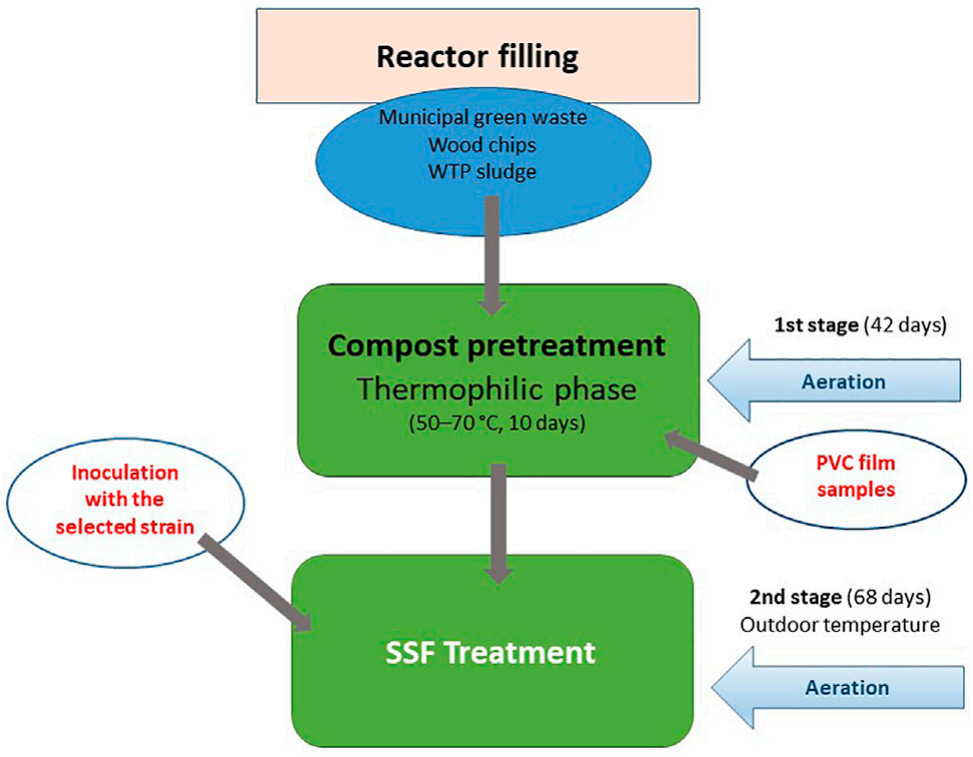
Schematic diagram of two-stage pilot scale, aerobic degradation of PVC films carried out in Janites KS1.2 and AK 2.0 reactors. For details *see* section 2.4.

The SSF process combined a controlled composting phase that served for a pretreatment of the virgin PVC material and a following biodegradation process when the compost material containing the PVC samples, resulting from the previous stage, was inoculated by a fungal or bacterial strain tested (Figure 1). The solid phase consisted of a wastewater treatment plant sludge (WTP, Teva Czech Industries, Opava, Czech Republic), solid municipal green waste and bush- and small-tree branches chipped into small pieces (Supplementary Figure S2). The process was started in the first step by composting of the material in which the PVC samples were buried. The first step included a thermophilic phase during which the inner temperature was maintained at 50–70°C for 10 days, followed by mesophilic and maturation phases taking about 3 weeks, when the material cooled down to 30–40°C. The process was controlled by aeration according to a computer program developed by Janites, s.r.o. (Havířov, Czech Republic) using the optimal composting curve. The temperature and humidity inside the composted material were measured daily. The PVC films wrapped in nylon mesh were inserted in the compost material before the composting process was started. At the beginning of the second step, the selected fungal or bacterial strains were added in the form of a homogenized, liquid-medium inoculum (7-day-old MEG culture, 2,500 ml) in the case of the Janites KS 1.2 reactor or in the form of a wheat bran-grown inoculum (3-week-old, 2,500 g, moisture 50%) amended with a salt solution (cf. section 2.3) in the case of the Janites AK 2.0 reactor. The inocula were thoroughly mixed with the composted material. The degradation phase took place at outdoor temperature. The experiment was carried out in the period of mid-May-mid-September in order to ensure convenient outdoor temperatures, the experiment was terminated when the outdoor temperate dropped to 15°C. In the controls, no microorganism was added in the second step.

Two types of pilot-scale reactors were used in the biodegradation process. In a Janites KS 1.2 reactor (Janites s.r.o., Havířov-Prostřední Suchá, Czech Republic, Supplementary Figure S1), the composting and subsequent SSF degradation were carried out in polyethylene bags (PE) 1 m long, filled with the compost mixture and aerated with an inside aeration tube. The controlling factor was the amount and frequency of aeration by a compressor that controlled the inside temperature and humidity, that were daily monitored using stabbing probes, a thermometer and a hygrometer connected to a COMET S3631 recorder (Micronix, spol s r.o., Prague, Czech Republic). The composting process was computer-controlled using a composting program (Janites, s.r.o., Havířov, Czech Republic). The number of bags connected to the device was six in maximum, the reactor worked in the conditions of the outdoor temperature and forced aeration ensured by an air compressor (Janites, s.r.o., Havířov, Czech Republic). The reactor operated in closed-system conditions.

The other reactor type used was a Janites AK 2.0 reactor (Janites s.r.o., Havířov-Prostřední Suchá, Czech Republic, Supplementary Figure S2) where the degradation process was realized in a two-chamber, horizontal, rotating drum system. The rotation speed controlled the aeration of the system and the inside humidity. The reactor consisted of a metallic cylinder mounted horizontally on a stand that was divided into two 1 m^3^ chambers. Solids were separately filled in the chambers that worked in a closed system mode and could thus be used as two reactors running in parallel. The controls and controlling systems were the same as in the case of the reactor Janites KS 1.2. The reactor was working at the outdoor temperature and the aeration was ensured by air diffusion depending on the speed of drum rotation that was operated by an electric drive fuelled by a 12 V-battery. The inoculation of the two reactor chambers was separate using a solid-state fungal inoculum with either a volume of 5 L of a homogenized liquid-medium microbial culture or with 2.5 kg of a solid-state fungal inoculum.

Formulation of the solid phase for the first stage was controlled by KOMPOST program (Janites s.r.o., Havířov-Prostřední Suchá, Czech Republic) and is shown in Table 1 and Supplementary Figure S3. The final values before the composting process was started were C:N 33.6 and humidity 58.0%.

**TABLE 1:** Formulation of the compost solid phase.

Solid phase material	C/N ratio	Humidity (%)	Amount (kg)
WTP sludge	21.7/3.1	94	7
Municipal green waste	57.8/3.4	82	63
Chipped wood	53.0/1.0	15	41

### Scanning Electron Microscopy

A JSM-6610LV scanning electron microscope (JEOL Ltd., Peabody, MA, United States) was used in the secondary imaging electron mode for the assessment of changes of the surface of PVC films surface treated by microorganisms. The samples were fixed with 3% glutaraldehyde, dehydrated through alcohol series, critical-point-dried, and putter-coated with gold. Then they were analysed in the above scanning electron microscope using the following conditions: accelerating voltage 17 kW, spot size 30, working distance 22 mm.

### Fourier Transform Infrared Spectroscopy

All studied polymers were analysed in the mid-infrared region using infrared spectroscopy. A Nicolet 6700 FTIR spectrometer (Thermo scientific, United States) equipped with KBr beamsplitter and DTGS/KBr detector was used. The samples were measured using the attenuated total reflectance (ATR) method with single bounce diamond crystal. A total of 64 scans with a resolution of 4 cm^−1^ were collected for each spectrum. The spectra were collected in the region 4,000–400 cm^−1^. Each sample was measured three times and the spectra were averaged.

### Mutagenicicty Tests

Mutagenicity of culture liquids obtained by 60-day exposure of virgin PVC to fungal and bacterial strains in liquid Czapek-Dox and Bushnel-Haas media, respectively, was measured using the Ames test and SOS Chromotest. In the Ames test (OECD, 2020; Mortelmans and Zeiger 2000), a plate-incorporation version of the *Salmonella typhimurium* His^−^ reversion assay was used, with or without the *in vitro* metabolic activation with the rat liver S9 microsomal fraction and cofactor mixture. The auxotrophic strains TA100 and TA98 were used for detection of the base substitution mutations and frameshift mutations, respectively. The mutagenic activity was expressed as a number of revertant colonies (Rt) obtained with the treated sample compared to the number of revertant colonies obtained with the control sample (Rc). The mutagenicity index was calculated as a ratio of Rt/Rc and a twofold increase of the index was considered to be significant. Mutation potential represented the mutagenicity index for the concentration of the compound tested that was read from the linear region of the dose-response curve (Ames et al., 1975). Each test was repeated at least three times using two replicate plates for each sample and the results were calculated using SALM software (Broekhoven and Nestmann, 1991).

The SOS Chromotest (Quillardet et al., 1982), a quantitative bacterial assay for genotoxins based on an induction of SOS function by these compounds, used the *E. coli* K12 strain PQ37. The mutagenic activity of the samples was determined by the ratio of the inducible ß-galactosidase activity to the constitutively-synthesized alkaline phosphatase. The induction factor for a compound at a certain concentration was defined as a ratio Rc/Ro where Rc and *Ro* were the respective mutagenic activities measured in the presence and absence of the compound tested. The SOS-inducing potency (SOSIP) was a single parameter representing the induction factor per concentration of the tested compound. SOSIP was determined from the linear region of a dose-response curve (Quillardet and Hofnung, 1985). The Rc/Ro value of 0.5 or more was considered to be significant and demonstrated a positive mutagenic effect.

## Results and Discussion

### Selection of Strains in Liquid-Medium and SSF Conditions

Before used in a two-stage degradation in compost and the SSF culture, the strains were tested for the ability to attack PVC material in liquid media, namely the fungal strains in a Czapek-Dox medium pH 6.8 and the bacterium *B. amyloliquefaciens* in a Bushnel-Haas medium pH 7.0 (Table 2). Maximal mass reductions of virgin PVC films measured gravimetrically obtained with *T. hamatum*, *T. suaveolens* and *P. ellipsoidea* were in the range of 18–20% after a 60-day treatment. The mass reduction caused by the attack of *B. amyloliquefaciens* was slightly lower (Table 2). The abiotic controls showed mass reduction values of 9–10%, probably due to the leaching of additives (Kastner et al., 2012; Suhrhoff and Scholz-Bottcher, 2016). The mass reduction values obtained were higher than those reported by Webb et al. (2000) for various fungal organisms in a mineral medium where the weight loss values ranged from 1.4 to 6.8% after 6 weeks at 25°C. Similarly, 5.7–11.7% weight reductions of PVC films serving as major carbon and energy source by marine anaerobic consortia after 24 months were documented (Giacomucci et al., 2020) whereas a 13.1 ± 0.4% virgin PVC weight decrease was reported after an exposure to *Pseudomonas citronellolis* for 90 days at 30°C (Giacomucci et al., 2019).

**TABLE 2:** Mass-reduction of PVC film by new isolated strains in liquid medium after 60 days at 28°C.

Microorganisms	PVC mass reduction, %
Fungi	
*Trichoderma hamatum*	19.9 ± 0.4
*Byssochlamys nivea*	15.5 ± 0.9
*Trametes suaveolens*	18.3 ± 0.7
*Pseudallescheria ellipsoidea*	18.3 ± 0.7
*Penicillium olsonii*	9.0 ± 0.7
*Penicillum variabile*	11.5 ± 3.3
*Scopulariopsis brevicaulis*	7.1 ± 0.7
*Aspergillus caesiellus*	8.7 ± 0.3
*Aspergillus terreus*	10.1 ± 1.7
*Graphium* sp	10.5 ± 3.9
Abiotic control	9.9 ± 2.9
Bacterium	
*Bacillus amyloliquefaciens*	17.5 ± 0.2
Abiotic control	8.8 ± 0.5

PVC contains large amounts of additives, mostly phthalate esters, and chlorine atoms (Glas et al., 2014; Wen et al., 2014). Phthalate esters have been proven to exhibit endocrine disrupting and genotoxic effects (Al-Saleh et al., 2017; Sicinska et al., 2021). Similarly, chlorine and its compounds, when reacting with organic molecules, can produce compounds with genotoxic effects (Richardson et al., 2007; Rincon-Bedoya et al., 2013). Therefore, genotoxicity of culture liquids obtained by a 60-day exposure of virgin PVC films to fungal and bacterial strains listed in Table 2 were measured using the Ames test and SOS Chromotest. No genotoxicity was detected in the culture liquids obtained after the exposure of PVC films to any of the microorganisms used.

*T. hamatum*, *A. terreus* and *B. amyloliquefaciens* were chosen for a test in the SSF culture because of their efficient attack of virgin PVC films in the liquid-medium and a robust growth under various conditions. In addition, two other fungal strains, *T. abietinum* and *S. apiospermum* and a strain of bacterium *R. ruber*, that were isolated during the study, were also included in the SSF biodegradation tests. The results measured in wheat bran-based SSF cultures showed mass reduction values of 7–9%, the highest values were obtained with *A. terreus* (8.6 ± 0.3%), *T. hamatum* (8.3 ± 0.5%) and *B. amyloliquefaciens* (9.2 ± 0.3%) (Table 3). The weight losses were about two times lower compared to a weight loss of a PVC cable exposed to a consortium of microorganisms from a biowaste fermentation plant under anaerobic landfill simulation conditions for 28 months that were caused by the removal of plasticisers (Mersiowsky et al., 2001). The weight loss was much faster in our SSF cultures as Mersiowski and co-workers (2001) did not observe any until 15th month of incubation.

**TABLE 3:** Mass-reduction of PVC film by new isolated strains under SSF conditions after 60 days at 28°C.

Microorganisms	PVC mass reduction, %
Fungi	
*Aspergillus terreus*	8.6 ± 0.3
*Trichoderma hamatum*	8.3 ± 0.5
*Trichaptum abietinum*	7.2 ± 1.1
*Pseudallescheria ellipsoidea*	5.7 ± 2.4
Bacteria	
*Bacillus amyloliquefaciens*	9.2 ± 0.3
*Rhodococcus ruber*	7.7 ± 0.9
Abiotic control	7.7 ± 0.1

Lower PVC films mass reduction values obtained in SSF conditions, compared to the liquid-medium conditions, could be explained by a better mass transfer in liquid phase, compared with the solid phase in the SSF culture (Stotzky and Burns, 1982). In the liquid phase, the accessibility of PVC film surface to extracellular enzymes produced by the microorganisms is better as the enzymes are distributed in the liquid medium whereas in the solid phase, they are more localized, which may hinder their contact with the PVC film surface (Novotny et al., 2004).

**FIGURE 2:**
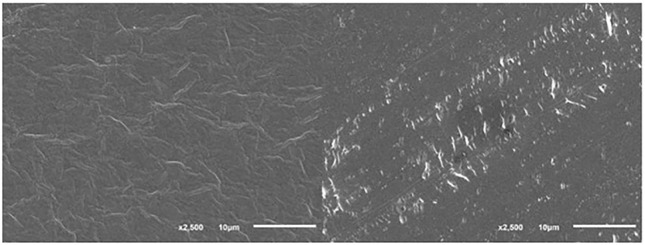
PVC film surface before (left) and after (right) biodegradation in the presence of *B. amyloliquefaciens* under SSF conditions; SEM conditions: SEI, WD 22, SS 30, 17 kV.

The PVC films after degradation under SSF conditions were scanned by SEM to show whether the surface was changed by the exposure to the microorganisms. An example can be seen in Figure 2 where a slightly different structure of the PVC film surface was recognized before and after the 60-day treatment with *B. amyloliquefaciens*. Efficient colonization of the PVC film surface by *B. amyloliquefaciens* and other microorganisms was observed in our experiments. In order to measure the weight reduction, the bacterial and fungal colonies had to be removed by the effect of SDS, sonication and intense washing with ethanol and distilled water (Sivan et al., 2006). We cannot exclude that certain changes of the PVC film surface could also be caused by this washing procedure. A number of studies documented that the colonization of plastic surfaces by microbial biofilms often resulted in their damage and is considered to be the first step in the microbial attack (Gu, 2007). Colonization by bacteria of the *Bacillus* genus resulting in an increased surface roughness accompanied by a weight loss was observed in the case of PE plastic (Nowak et al., 2011).

### Aerobic Degradation of PVC Films in a Combined, Two-Stage Pilot-Scale Process

The selection of strains for the two-stage degradation process combining the compost-pretreatment and SSF biodegradation included the fungi *A. terreus*, *T. hamatum*, *T. abietinum P. ellipsoidea* and the bacterium *B. amyloliquefaciens* that exhibited high PVC film mass reduction rates in the liquid medium- and SSF cultures. The pilot-scale biodegradation experiments were realized using Janites KS 1.2 and AK 2.0 reactors. The pretreatment of virgin PVC films by the composting process took 42 days, then the compost was augmented by massive inoculation with a selected microorganism, and the degradation continued under SSF conditions for further 68 days. During both stages the former reactor was kept under forced aeration by a compressor whereas in the latter one the aeration was ensured by air diffusion depending on the drum rotation. The first collection of PVC samples was after the end of the composting phase and the second one at the end of the SSF phase. Hence, the total length of the period when PVC films were exposed to the microbial attack was 110 days, first to the native microbiome of compost microorganisms acting during the first stage and, subsequently, to a combined attack by the native compost microbiome and the microorganism added in the second stage.

A significant PVC film mass reduction occurred already during the first compost phase, ranging between 8 and 11% in KS 1.2 reactor. Further weight decrease of the PVC samples was observed at the end of the subsequent SSF treatment after the culture was amended by inoculation with a selected microbial strain. When *T. hamatum* or *B. amyloliquefaciens* were added to the SSF cultures, the total PVC film mass reductions attained 29.3 ± 19.8 and 33.2 ± 22.2%, respectively, surpassing the values that were obtained after a 30-day incubation with *P. citronellolis* in a liquid medium (14%, Giacomucci et al., 2019) or in a landfill-simulation experiment after 28 months (14%, Mersiowsky et al., 2001). However, FTIR measurements (*see* section 3.3) documented that the weight reductions probably represented degradation and removal of molecules of the additive rather than of the PVC polymer, which was in keeping with other reports (e.g. Mersiowsky et al., 2001; Giacomucci et al., 2019; Ru et al., 2020). Sun et al. (2021) composted PVC microplastics for 60 days to observe a carbon loss of 17% and an oxygen increase of 3% due to biological oxidation resulting in the formation of oxygen-containing functional groups, namely the alcohol hydroxyl group. Such an increase in the amount of oxygen-containing functional groups is similar to the effect of various pretreatment procedures (e.g. Chiellini et al., 2006; Novotny et al., 2018) and can improve the biodegradability of the plastic (Chen et al., 2020). However, the FTIR analysis showed that the pretreatment by composting did not make PVC polymer susceptible to the attack by the microorganisms. Unfortunately, GPS analysis was not available and thus the plastic depolymerization pattern resulting from the biodegradation, as described by Wu and Criddle (2021), could not be established.

Other microorganisms tested (Table 4) were able to increase the weight reduction in the second stage much less than *T. hamatum* and *B. amyloliquefaciens*, when added for augmentation. Comparably, low PVC weight losses were reported with various micromycetes and yeasts (1.8–6.8% after 6 weeks at 25°C; Webb et al., 2000) or with anaerobic marine consortia (up to 11.7 ± 0.6% after 7 months at 20°C; Giacomucci et al., 2020).

**TABLE 4:** Mass-reduction of PVC film during the compost pretreatment and a subsequent treatment with selected fungal and bacterial strains under SSF conditions in KS 1.2 and AK 2.0 reactors.

Organism	After compost pretreatment% (W/W)	After subsequent SSF treatment with selected strain% (W/W)
Janites KS 1.2 reactor		
*A. terreus*	9.9 ± 0.1	11.3 ± 0.7
*T. hamatum*	10.3 ± 0.6	29.3 ± 19.8
*T. abietinum*	11.4 ± 0.5	10.8 ± 0.2
*P. ellipsoidea*	9.0 ± 0.5	10.7 ± 0.3
*B. amyloliquefaciens*	8.8 ± 2.1	33.2 ± 22.2
Control	8.5 ± 0.7	10.0 ± 0.4
Janites AK 2.0 reactor		
*A. terreus*	10.8 ± 0.6	14.2 ± 5.8
*T. hamatum*	16.9 ± 7.9	16.3 ± 3.5
Control	8.2 ± 0.4	9.6 ± 0.3

In AK 2.0 reactor where aeration was ensured by drum rotation, two fungi, *T. hamatum* and *A. terreus*, were tested to investigate the effect of shear forces and less intensive aeration on the effectivity of biodeterioration of PVC films. When the latter fungus was added in the second stage the cumulative weight reduction reached 14.2 ± 5.8%, compared to that of 11.3 ± 0.7% obtained in the aerated bags of KS 1.2 reactor. However, in the case of *T. hamatum*, no weight decrease occurred in the second stage, the total PVC film mass reduction was 1.8 times lower compared to the aerated-bag-reactor process (Table 4). We may speculate that the conditions for *T. hamatum* in KS 1.2 reactor that included a static culture and forced aeration were more favorable compared to the exposure to shear forces and aeration only by air diffusion in the AK 2.0 reactor. This effect was not observed with *A. terreus* (Table 4). Mycelia of some filamentous fungi have been shown to be sensitive to shear forces caused by friction (Gong and Zhong, 2005; Tang et al., 2007).

High standard deviation values measured with some microorganisms (Table 4) can be attributed to both the fragmentation of PVC films into small pieces that were not easy to collect and/or small amounts of microbial biofilms that could remain attached to the plastic surface even after washing (Briassoulis et al., 2015; Malachova et al., 2020).

### FTIR Analysis

**FIGURE 3:**
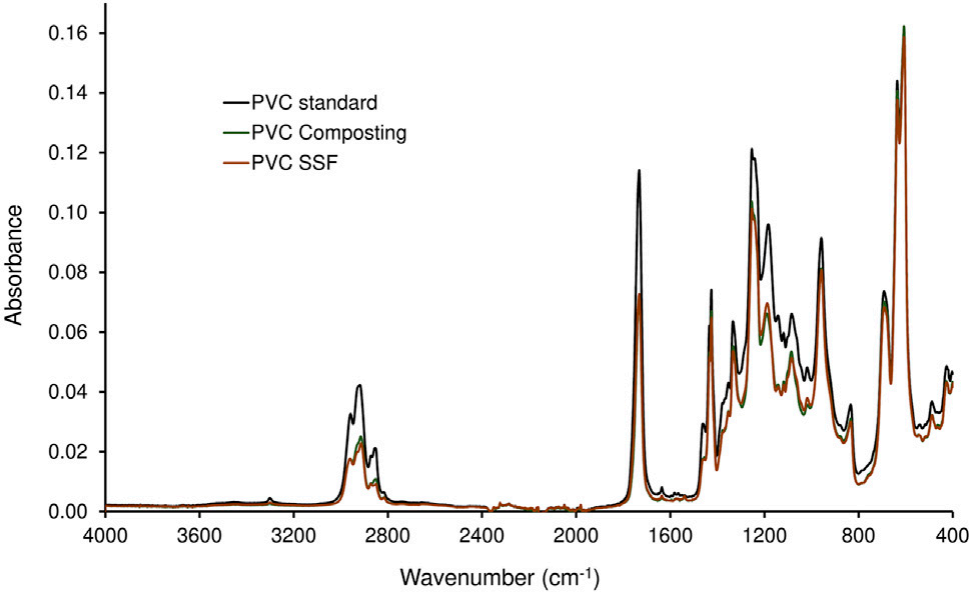
Spectra of PVC treated in a two-stage degradation procedure by composting (first stage, 42 days; PVC Composting) and by *B. amyloliquefaciens* (second stage, 68 days; PVC SSF) compared to virgin PVC standard.

**FIGURE 4:**
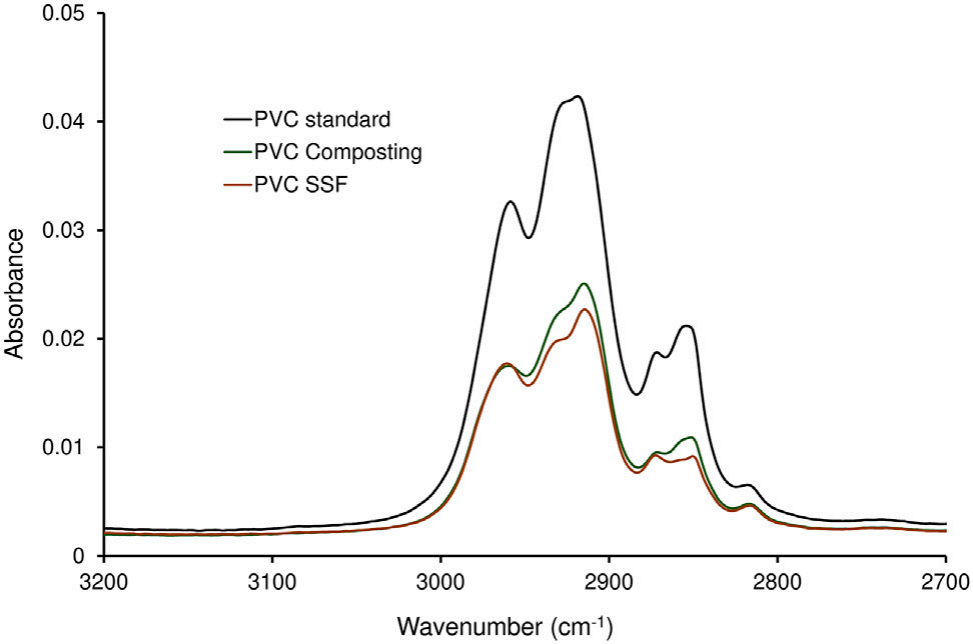
Spectra of C-H stretch vibrations region of PVC treated in a two-stage degradation procedure by composting (first stage, 42 days; PVC Composting) and by *B. amyloliquefaciens* (second stage, 68 days; PVC SSF) compared to virgin PVC standard.

The PVC samples collected after a two-stage degradation process in the KS 1.2 reactor using *B. amyloliquefaciens* in the second stage, where the largest weight reduction was measured (Table 4), were analyzed by FTIR (Figures 3, 4). The spectra were compared with the PVC standard before biodegradation. The used material was commercially available foil, therefore the bands of PVC as well as of the additives were present in the spectrum. Spectrum of pure PVC without additives contains only bands between 3,000 and 2,800 cm^−1^ which can be assigned to the stretch vibration of C–H bonds, bands around 1,430 cm^−1^ which belong to the–CH_2_– deformation vibrations, and a band around 605 cm^−1^ which can be assigned to C–Cl stretch vibration. The spectrum in Figure 3 included other bands as well which probably belonged to additives. Especially the band around 1,730 cm^−1^ which can be assigned to the stretch vibration of carbonyl group (C=O). Carbonyl is not present in the PVC molecule but it is part of the most common plasticizers used in PVC such as phthalates or adipates. Bands in the region 1,200–900 cm^−1^ belonged probably to the additives as well. Moreover, additives are often organic compounds and they contribute to the absorbances of bands between 300 and 2,800 cm^−1^, too. A decrease of the bands’ absorbances occurred mainly in the regions of C–H stretch vibrations (3,000–2,800 cm^−1^), C=O stretch vibrations (1,730 cm^−1^), C–H and N–H deformation vibrations (1,500–1,300 cm^−1^), and C–O stretch and skeletal vibrations (1,300–800 cm^−1^). However, a negligible decrease was observed for the band of C–Cl stretch vibration after the biodegradation process. Bonds C–Cl are present only in the PVC polymer itself and they were not affected during biodegradation. Therefore, the conclusion was that the biodegradation affected primarily the additives and the degradation of PVC was negligible (Socrates, 2007). The shape of bands in the region of the C–H stretch vibrations (Figure 4) varied as well. The change of the bands’ shape supported the conclusion affirming the preferential degradation of additives in the studied material because C-H bonds were present both in PVC and the additives.

The plasticizers most often used in PVC are phthalic acid esters whose biodegradability by various bacteria and fungi has been well proven (e.g. European Commission DGXI.E.3, 2001; Amir et al., 2005; Liang et al., 2008; Chang et al., 2009). As the type and amount of plasticizers used in PVC films were not indicated by the producer, their formulation being confidential, and the relevant analytical method was not available, we had to rely in the discussion on indirect evidence concerning the removal of the additives during biodegradation. Despite a high mass reductions of PVC films obtained by the combined attack by compost microorganisms and *B. amyloliquefaciens* (Table 4), the PVC polymer chains were not degraded, as evidenced by FTIR. However, FTIR analysis confirmed the presence of bands characteristic of plasticizers used in PVC materials whose shape was changed during biodegradation (Figure 2). Consequently, the conclusion was that the massive biodeterioration of the plastic material that occurred, most probably resulted from the biodegradation of additives in first and second stages (cf. Mersiowsky et al., 2001; Ru et al., 2020).

A pretreatment of plastics can result in a formation of functional groups that increase the plastics susceptibility to microbial attack. It was evidenced for PE plastics exposed to thermal oxidation carried out at temperatures ranging from 55 to 80°C (e.g. Volke-Sepulveda et al., 2002; Manzur et al., 2004; Corti et al., 2010). Here the PVC films were pretreated in biooxidative conditions at a high temperature ensured by the composting process that included a thermophilic phase of 60–70°C, where the plastic was also exposed to the action of the compost microorganisms. However, the pretreatment by composting did not make the PVC polymer prone to microbial attack as evidenced by FTIR analysis. This is in agreement with the observation of Pardo-Rodriguez and Zorro-Mateus (2021) who compared the biodegradation of heat-treated- (150°C, 1 h) and virgin PVC by *Penicillium* sp. and *Mucor* sp. fungi and found no difference between the pretreated and virgin PVC.

The results indicated that not even a combination of the highly degradative actions of the composting process and bacterial and fungal strains with broad biodegradation activities were able to degrade the recalcitrant PVC polymer (Zafra and Cortes-Espinosa, 2015; Meng et al., 2019; Yuan et al., 2019; Davolos et al., 2021). Further characterization of depolymerization of PVC material is needed to understand the patterns describing the extent of PVC depolymerization during the two-stage process, as observed during plastics degradation by other researchers (Wu and Criddle, 2021). Future research should also focus on determination of chlorine release during the treatment to find out whether mineralization of PVC polymer occurred (Peng et al., 2020).

## Conclusion

The resistance of PVC to biodegradation makes it a matter of concern with respect to the environmental pollution due to its high production and wide industrial applications. Compared to other petroleum-based plastics, PVC contains much higher percentage of additives, mostly phthalates, that are biodegradable and have been recognized to be endocrine disruptors. In our case study, virgin PVC films were treated in a two-stage process that included, first, a pretreatment by biological oxidation at a high temperature by compost microorganisms and, second, an exposure to selected strains of fungi and bacteria isolated from the surface of composted plastics. Gravimetric measurements documented significant weight losses both in the first and second biodegradation stages when *T. hamatum* and *B. amyloliquefaciens* were used for augmentation but FTIR analysis detected no biodegradation of PVC polymer. Comparably to other studies only the additives were removed to diminish the mass of PVC films in both stages of the biodeterioration process. Further research is needed to characterize the enzyme activities capable to depolymerise PVC polymer whose existence was revealed in some recent studies.

## Data Availability

The original contributions presented in the study are included in the article/Supplementary Material, further inquiries can be directed to the corresponding author.
