# Supplementary material for: Biodeterioration of Compost-Pretreated Polyvinyl Chloride Films by Microorganisms Isolated From Weathered Plastics
Source: Front Bioeng Biotechnol. 2022 Feb 10;10:832413. doi: 10.3389/fbioe.2022.832413 (PMC8867010; doi:10.3389/fbioe.2022.832413)
Supplement: Supplementary file 1 [file DataSheet1.docx]

Supplementary Material

# Supplementary Figures and Tables

## Supplementary Figures

**Supplementary Figure 1S**


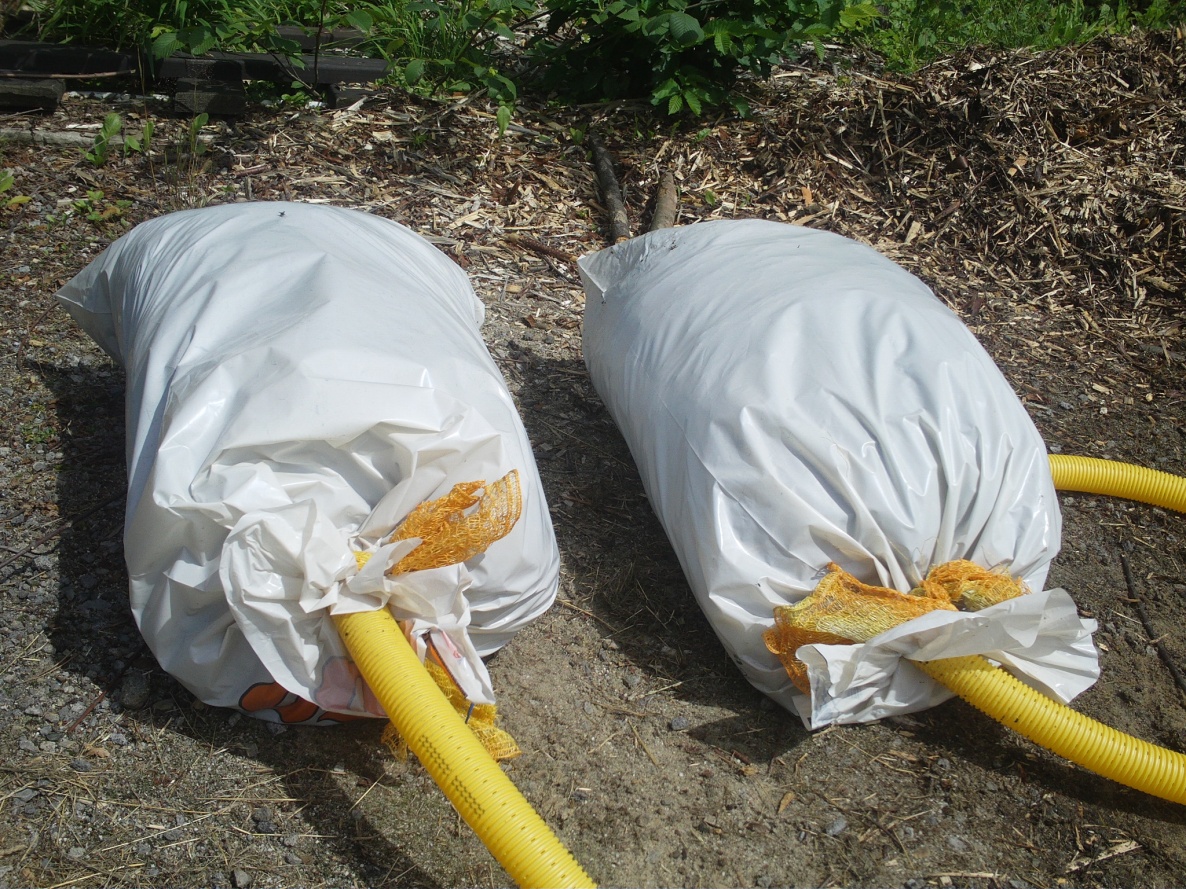


Fig. 1S Reactor Janites KS 1.2 (Janites s.r.o., Czech Republic) used for composting and subsequent SSF biodegradation after augmentation with a selected microbial strain. Reactor consisted of PE bags (length 1m) filled with a compost mixture, aeration was with an inside aeration tube connected to a compressor. The reactor worked at the outdoor temperature.

**Supplementary Figure 2S**

**
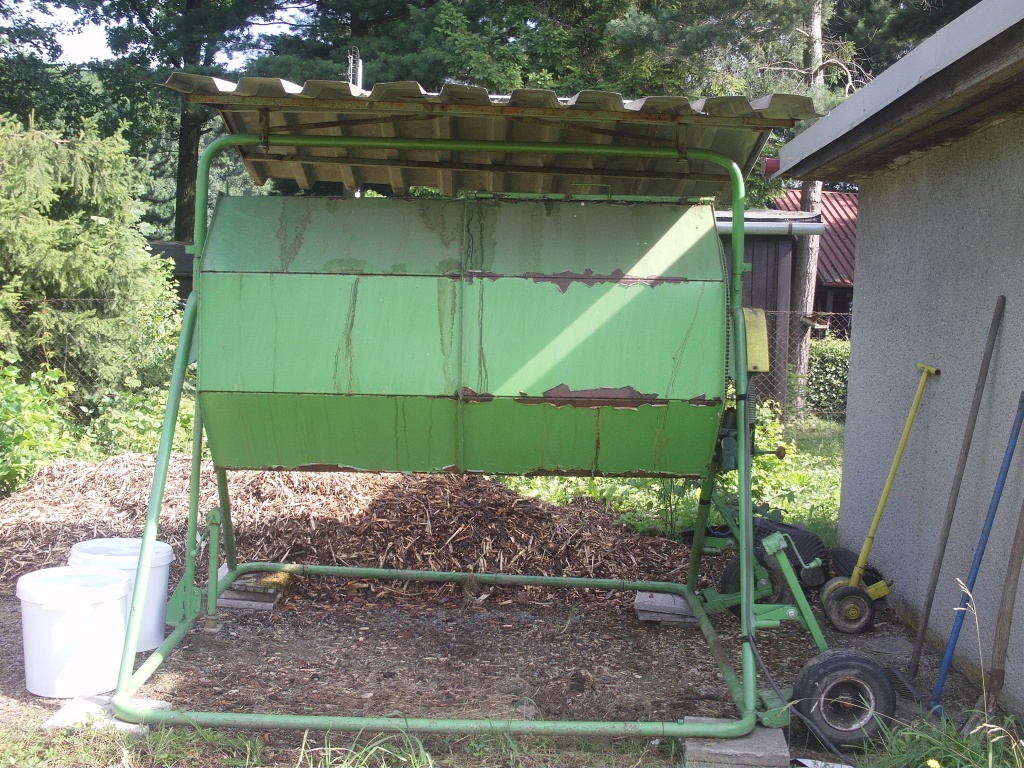
**

Fig. 2S Reactor Janites AK 2.0 (Janites s.r.o., Czech Republic) used for composting and subsequent SSF biodegradation after augmentation with a selected microbial strain. Reactor consisted of a two-chamber, horizontal, rotating-drum system: two independent chambers filled with compost solids, the volume of each chamber was 1m^3^, aeration was by air diffusion ensured by drum rotation. The reactor worked at the outdoor temperature.

**Supplementary Figure 3S**


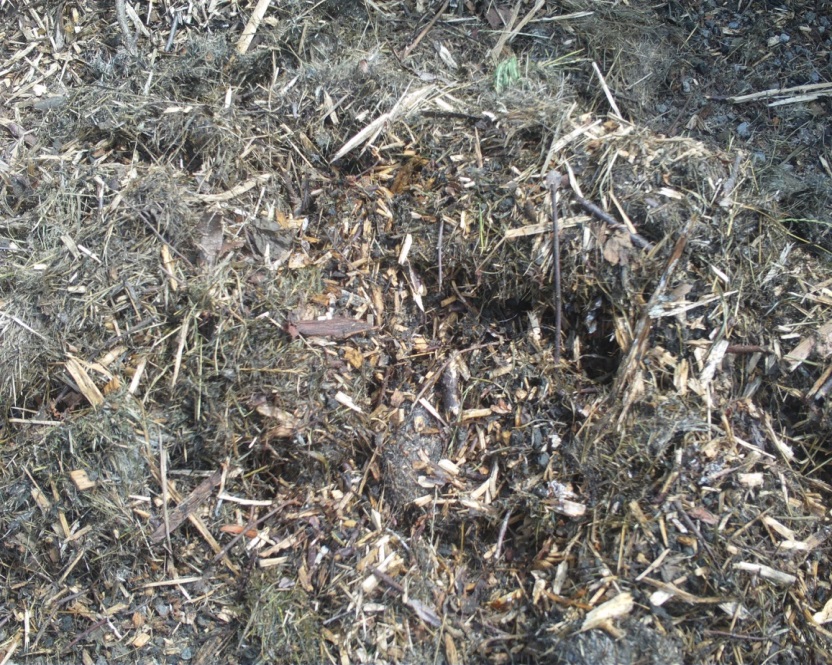


Fig. 3S Solid compost mixture used in the two-stage biodegradation process was composed of municipal green waste, chipped bush and tree branches (see Table 1); the composition was controlled by KOMPOST program, initial values: C/N 33.6%, humidity 58.0% (W/W).

**
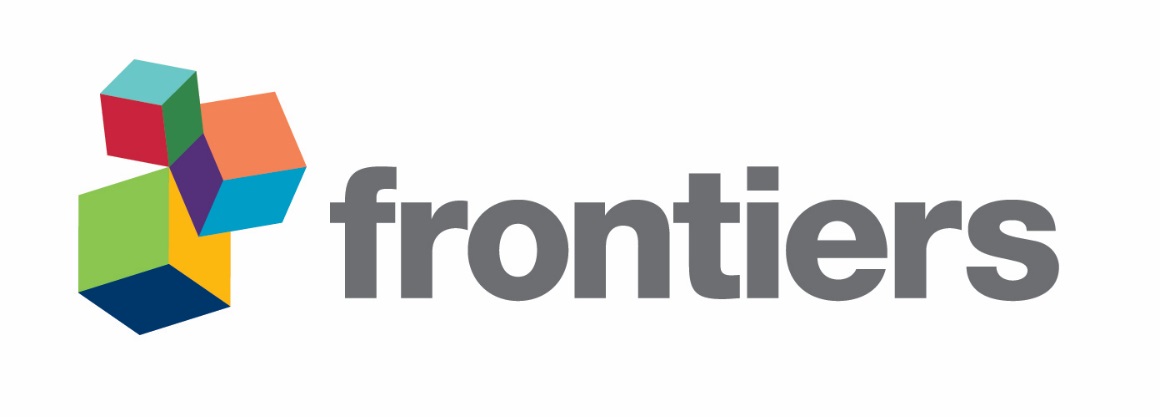
**
